# Hybrid Au@alendronate nanoparticles as dual chemo-photothermal agent for combined cancer treatment

**DOI:** 10.3762/bjnano.9.273

**Published:** 2018-11-27

**Authors:** Anouchka Plan Sangnier, Romain Aufaure, Laurence Motte, Claire Wilhelm, Erwann Guenin, Yoann Lalatonne

**Affiliations:** 1 Inserm U1148, LVTS, Université Paris 13, Sorbonne Paris Cité, Bobigny, France; 2 Laboratoire Matière et Systèmes Complexes, CNRS and University Paris Diderot, Paris, France; 3 Sorbonne Universités, Université de Technologie de Compiègne, Integrated Transformations of Renewable Matter Laboratory (EA TIMR 4297 UTC-ESCOM), Compiègne, France; 4 Service de Médecine Nucléaire, Hôpital Avicenne Assistance Publique-Hôpitaux de Paris, Bobigny, France

**Keywords:** alendronate, bisphosphonate, cancer treatment, gold nanoparticles, photothermia

## Abstract

A gold therapeutic nanoplatform with the same molecule used as reductant, coating and therapeutic agent has been developed in a one-pot, one-phase process using alendronate, a drug from the bisphosphonate family known for its antitumor effects. In addition, the core made of gold nanoparticles (NPs) brings thermal functionalities under irradiation within the first biological window (650–900 nm). The Au@alendronate nanoplatform thus provided a combined antitumor activity through drug delivery and photothermal therapy. Au@alendronate NPs inhibited in vitro the proliferation of prostate cancer cells (PC3) in a dose-dependent manner, with an IC_50_ value of 100 µM. Under NIR irradiation a temperature increase was observed leading to a reduction of the IC_50_ value to 1 µM, with total tumor cell death at 100 µM.

## Findings

Bisphosphonates (BPs) are used in the treatment of a variety of bone diseases, such as osteoporosis, solid tumor bone metastases and myeloma bone disease [1–4]. BPs contain two phosphonate groups linked by a common carbon atom (P–C–P) binding divalent metal ions (Ca^2+^, Mg^2+^, and Fe^2+^) by coordination of the two phosphonate groups. The BP affinity for calcium is improved by adding a hydroxy (–OH) group, for instance in HMBP (hydroxylmethylene bisphosphonate), allowing for a tridentate coordination to Ca^2+^ ions (Supporting Information File 1, Figure S4) and leading to a high affinity to bone (hydroxyapatite (Ca_10_(PO_4_)_6_(OH)_2_)) tissue [5–6]. We focus here on the antitumor activity of alendronate, a nitrogen-containing HMBP, clinically used as adjuvant (Fosamax^®^) in the treatment of prostate and breast metastatic cancers [7]. Nitrogen-containing HMBPs, such as alendronate, are inhibitors of the mevalonate pathway. They inhibit the prenylation of GTPase proteins, which affects cell morphology, replication and signalling that can cause cell death by apoptosis [8–9]. However, the in vivo therapeutic use of HMBPs is limited by low bioavailability. Once intravenously injected, free HMBPs are only slightly internalized by the cells and accumulates preferentially into bone tissue. Benyettou et al. showed that alendronate-coated magnetic NPs favour the intratumoral uptake and inhibit tumor growth [10].

HMBPs are also effective ligands to stabilize nanoparticles under biological conditions [11–15]. More recently, synthesis of gold and silver NPs have been developed using HMPB molecules [16–18]. For gold NPs, HMBPs act as both Au chelating and reducing agent comparable to citrates in the well-known Turkevich–Frens synthesis [19–20]. Besides, gold NPs exhibit a unique surface plasmonic resonance leading to strong enhancement of the absorption and scattering when exposed to electromagnetic radiation [21]. Due to this plasmonic absorption, light is converted to heat [22–26]. Photothermal therapy (PTT) is a powerful cancer-treatment technique. Gold NPs have to be activated within the biological transparency windows of 650–950 nm or 1000–1350 nm, to minimize light absorption by surrounding biological tissues [27]. The principal limitation of PTT is that it requires direct light irradiation, which reduces its effect against disseminated metastatic tumors. A promising strategy to increase the PTT efficiency is the combination with magnetic hyperthermia [28], or with chemotherapy [29–30].

Using a one-pot synthesis strategy, we developed Au@alendronate NPs for a combined application of the antitumor activity of alendronate and an efficient gold-mediated PTT. We further evaluated their combined chemo-photothermal antitumor activity.

### Synthesis and characterization of Au@alendronate NPs

**Figure 1:**
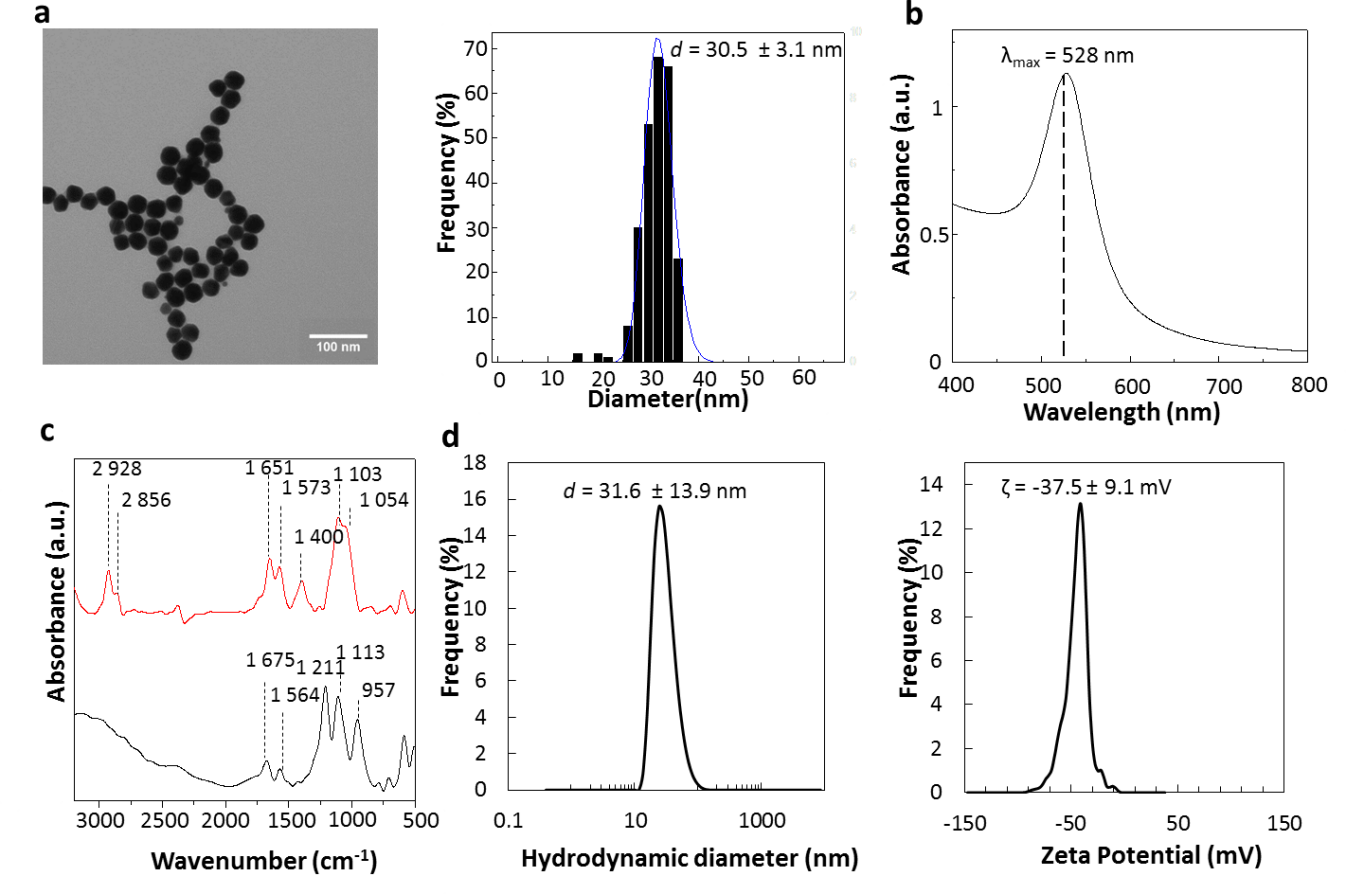
Au@alendronate NPs characterization: (a) transmission electron microscopy (TEM) image (left) and size distribution (right), (b) UV–vis spectrum, (c) FTIR spectra of Au@alendronate NPs (red curve) versus alendronate (black) curve, (d) Hydrodynamic diameter distribution (in volume, left) and zeta potential (right) of Au@alendronate NPs.

Gold NPs were synthesized in water by using alendronate both as reducing agent and chelating ligand. The gold solution is simply added to an alendronate solution at boiling temperature (see Supporting Information File 1). The excess of reactive species is eliminated by ultrafiltration. We thus obtained spherical NPs (Figure 1a, left) with an average diameter of 30.5 ± 3.0 nm (Figure 1a, right) and a plasmon band at 528 nm (Figure 1b).

Under similar synthesis conditions, gold NPs obtained with (1-hydroxy-1-phosphonopent-4-enyl)phosphonic acid (HMBPene), replacing alendronate were characterized by a smaller NPs size around 10 nm [16]. This indicates the influence of the reducing ligand on the crystal growth. Inductively coupled plasma atomic emission spectroscopy (ICP-AES) has been used to quantitatively determine the amount of alendronate per nanoparticles. 1.0 P atoms per 7.6 Au atoms was measured, which corresponds to 36,427 alendronate molecules per NP or a coating density of 12.5 alendronate molecules/nm^2^ for a 30.5 nm spherical gold NP. Remarkably, the alendronate density is much higher than estimated values for other NPs coated with HMBP molecules (i.e., 3.4 HMBPene/nm^2^) [18]. Indeed, alendronate is a zwitterion, capable of forming pairs of ions that generate multilayers around the gold NP.

The chemisorption of alendronate was qualitatively assessed (Figure 1c) by Fourier-transform infrared spectroscopy (FTIR) comparing the coated gold NPs (red curve) with free alendronate (black curve). Large modifications were observed within the PO region (900–1200 cm^−1^). The free alendronate spectrum exhibits two sharp peaks at 1211 and 957 cm^−1^, assigned to P=O and P–OH, respectively [31]. The broad band at 1113 cm^−1^ is characteristic for the vibrational mode for the PO_3_ group [32]. For Au@alendronate NPs the strong tightening of P=O and P–O vibration bands around 1000 cm^−1^ (red curve), which is characteristic of the chelation of phosphorus species on a metallic surface [11,16,33], suggests a coordination of phosphonates as chelating groups. The –NH scissoring (1564 cm^−1^) and bending vibration bands (1675 cm^−1^) for alendronate were slightly shifted in the Au@alendronate NPs confirming the multilayer formation through anion/cation interactions due to the alendronate zwitterion form. These results suggest that alendronate was grafted onto the nanocrystal surface through the phosphonate groups and could be activated for further biocoupling [10].

The excellent coating density led to a good colloidal stability, as confirmed by dynamic light scattering (DLS) measurements at physiological pH values. A hydrodynamic diameter equal to 31.5 ± 13.9 nm (Figure 1d) was determined, which is sufficiently close to the TEM crystal size to testify to the absence of gold NP aggregates. Au@alendronate NPs were stable at pH > 4.7 and at least four months after synthesis (see Supporting Information File 1, Figure S1). The negative zeta potential, equal to −37.5 ± 9.1 mV confirms the presence of alendronate on the surface providing negative charges, which allow colloid stabilization despite the presence of ammonium cations.

### Au@alendronate NPs as NIR photothermal nano-heater

**Figure 2:**
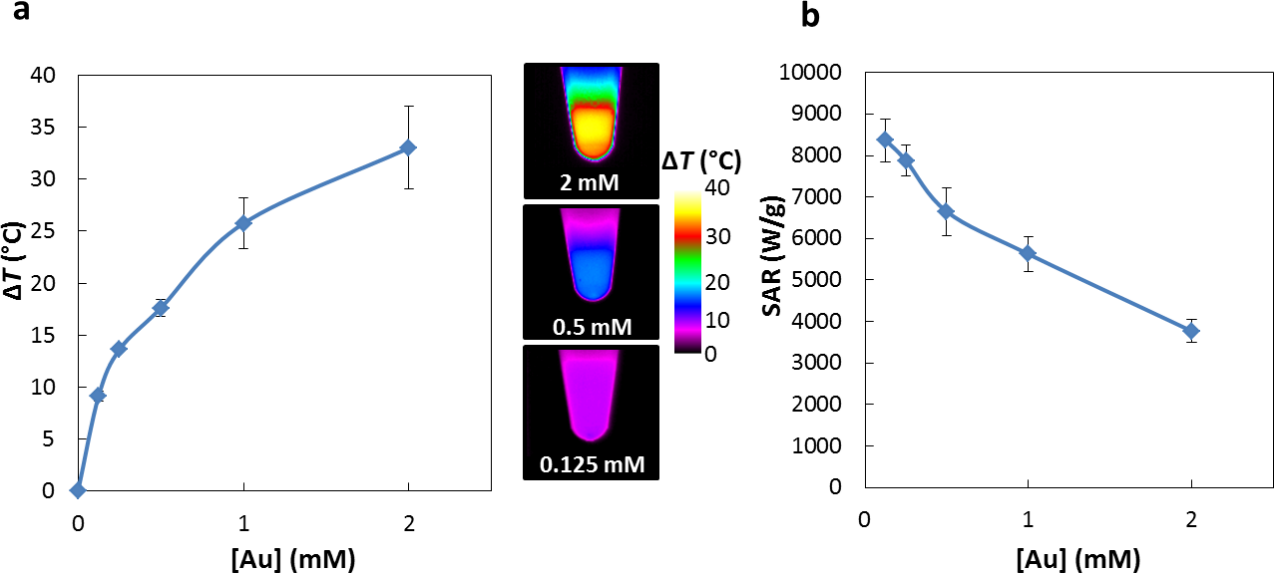
(a) Temperature increase and corresponding typical IR image and (b) SAR (W/g) as a function of the gold concentration under laser irradiation (1.7 W/cm^2^, 680 nm).

Since gold NPs bring their own therapeutic asset, in the form of PTT, we first evaluated the specific photothermal properties of Au@alendronate NPs. In cancer therapy, it is desirable to use NPs that are active in the near-infrared (NIR) region to minimize light absorption of the laser radiation by surrounding tissues [27,34]. The plasmonic absorption band of Au@alendronate NPs is centered at 528 nm, but there is still absorption above 800 nm (Figure 1b). Here, a laser operating at 680 nm and 0.3 W/cm^2^ (Supporting Information File 1, Figure S2) and 1.7 W/cm^2^ (Figure 2) was used. As we recently reported, this 1.7 W/cm^2^ laser power was efficient for inducing tumor growth inhibition in vivo without exhibit nonspecific phototoxicity [35]. In addition, this value is lower than the power used in many in vivo studies [36–38]. Figure 2 summarizes the heating characteristics of Au@alendronate NPs, measured in water as a function of the gold concentration. Figure 2 shows the plateau temperatures reached after 5 min of irradiation (Figure 2a), as well as the concentration-normalized heating efficiency expressed as the specific absorption rate (SAR) in watts per gram of Au (Figure 2b, see also Supporting Information File 1 for calculation details and Figure S2 for the temperature elevation at 0.3 W/cm^2^).

The temperature elevation clearly increased with the gold concentration and reached Δ*T* = 30 °C at a gold concentration of 2 mM. A temperature increase of Δ*T* = 9 °C is observed at a low gold concentration of 0.125 mM. The SAR is above 4000 W/g at high concentrations, reaching very high values (over 8000 W/g) at low concentrations. These photothermal properties are in good agreement with those of other thermal agents [23] and show the applicability of Au@alendronate gold NPs as potential photothermal agents.

The colloidal stability of Au@alendronate NPs at physiological pH values and their photothermal properties within the NIR first biological window allowed us to further consider their study in a biological environment.

### Au@alendronate NPs antitumor activity

**Figure 3:**
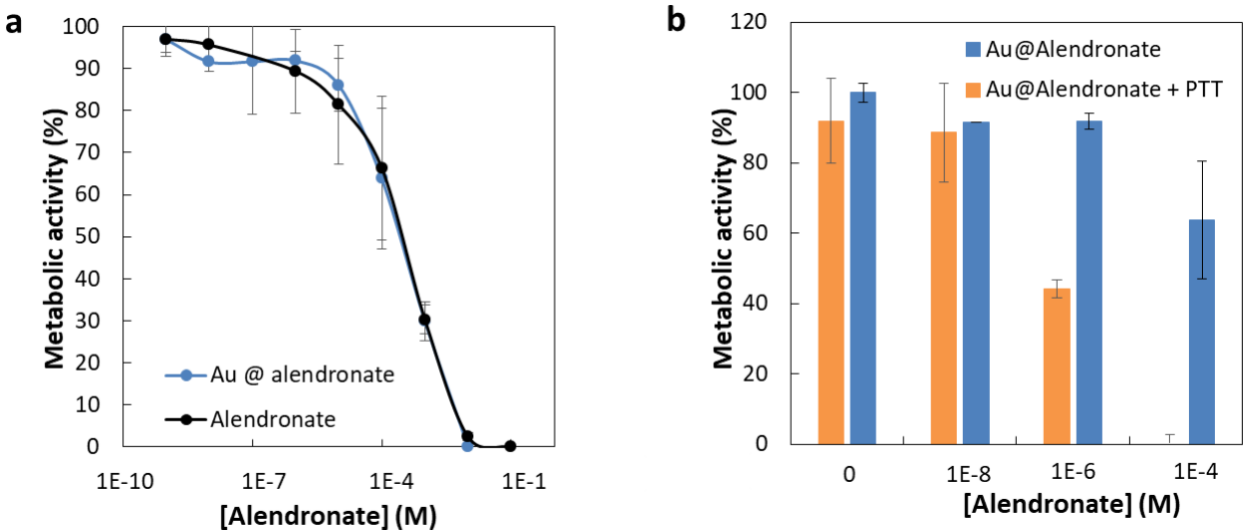
Metabolic activity of PC3 cells incubated (a) with free alendronate (black curve) and Au@alendronate NPs (blue curve), (b) with Au@alendronate NPs under the presence (orange column) or absence (blue column) of NIR irradiation (680 nm).

PC3 human prostate adenocarcinoma cells were selected to explore the potential of Au@alendronate NPs as antitumor agents [9]. PC3 cells were first treated both with free alendronate and with Au@alendronate NPs (at various extracellular alendronate concentrations from 1 nM up to 0.1 M) for 48 h. Metabolic activity (Figure 3) was determined by Alamar Blue assay (see Supporting Information File 1). With this assay the half maximal inhibitory concentration (IC_50_ value) can be determined. This value is a good indicator of the effectiveness of a compound for inhibiting biological or biochemical functions. Free alendronate and Au@alendronate gold NPs reduced cell viability in a concentration-dependent manner (Figure 3a) with an IC_50_ equal to 100 µM for both systems whereas Au@HMBP-PEG NPs [39] do not exhibit any cytotoxicity (Supporting Information File 1, Figure S3). Under similar cell-treatment conditions, this IC_50_ value is consistent with values obtained for free alendronate with other cancer cell lines [10]. More importantly, it indicates that Au@alendronate NPs perfectly retained the antitumor activity of alendronate suggesting the alendronate release within the intracellular environment. However, at relevant concentrations, complete cell death was not achieved. Hence, we included photothermal treatment by using a 680 nm laser calibrated to illuminate cells at 1.7 W/cm^2^. The metabolic activity on PC3 cells incubated with Au@alendronate NPs in presence or absence of laser irradiation is compared in Figure 3b.

At extracellular concentrations of alendronate below 1 µM, similar cell viability was observed in absence or presence of laser irradiation. This could be related to the low dose of internalized gold NPs and indicates that the laser power is sufficiently low to avoid nonspecific biological damage. At extracellular concentrations of alendronate over 1 µM, cell viability was considerably lowered in the presence of laser irradiation. The IC_50_ was reduced to 1 µM (instead of 100 µM), while at intermediate concentration of 100 µM, cell death was total. It clearly evidences the efficiency of the combined drug delivery and photothermal treatment of Au@alendronate NPs.

In summary, we developed a one-pot synthesis by simply mixing, in water, gold ions and alendronate molecules as reductant, coating and therapeutic agent. The synthesized Au@alendronate NPs maintain the alendronate antitumor activity, which is greatly improved under NIR laser radiation. These results pave the way for an efficient antitumor activity of Au@alendronate NPs through combining drug delivery in the form of a nanoplatform carrying alendronate and photothermal therapy. Indeed, Au@alendronate NPs will accumulate within cells because of the enhanced permeability retention effect: An enhanced permeability of blood vessels near the tumor allows for the penetration of nanoparticles into the tumor. The impaired lymphatic function within the tumor will not be able to clear those nanoparticles efficiently [40]. This proof-of-concept study will be completed by the intracellular behavior of Au@alendronate NPs with a special attention to alendronate release under photothermal activation.

## Supporting Information

File 1 Materials and methods and supplementary figures.
