# Supplementary material for: Hybrid Au@alendronate nanoparticles as dual chemo-photothermal agent for combined cancer treatment
Source: Beilstein J Nanotechnol. 2018 Nov 27;9:2947–52. doi: 10.3762/bjnano.9.273 (PMC6278761; doi:10.3762/bjnano.9.273)
Supplement: File 1 — Materials and methods and supplementary figures. [file Beilstein_J_Nanotechnol-09-2947-s001.pdf]

BEILSTEIN JOURNAL OF NANOTECHNOLOGY

Supporting Information

for

Hybrid Au@alendronate nanoparticles as dual chemo-photothermal agent for combined cancer treatment

Anouchka Plan Sangnier, Romain Aufaure, Laurence Motte, Claire Wilhelm, Erwann Guenin and Yoann Lalatonne

Beilstein J. Nanotechnol. **2018,** *9,* 2947–2952. doi:10.3762/bjnano.9.273

Materials and methods and supplementary figures

License and Terms: This is a supporting information file under the terms of the Creative Commons Attribution License (http://creativecommons.org/licenses/by/4.0). Please note that the reuse, redistribution and reproduction in particular requires that the authors and source are credited.
The license is subject to the *Beilstein Journal of Nanotechnology* terms and conditions: (https://www.beilstein-journals.org/bjnano)

Materials and methods

Alendronate synthesis

Alendronate was synthesized according to the general procedure for linear aliphatic BPs and characterized by ^1H and ^{31}P NMR [1,2]. BPs was prepared from the corresponding carboxylic acid precursor according to the following reaction carboxylic acid (150 mmol) and H_3PO_3 (150 mmol) were introduced in a three-necked round-bottom flask under inert atmosphere followed by 30 mL of methanesulfonic acid. After heating at 65 °C for 1 h, PCl_3 (40 mmol) was added slowly and the reaction allowed to proceed overnight at 65 °C. The resulting yellow viscous reaction mixture was cooled to room temperature, quenched with 500 mL of ice-cold water. The pH was adjusted to 4.3 with a NaOH aqueous solution (0.5 M) and the obtained white precipitate was collected by filtration. This solid was washed five times with a mixture of methanol/water (95:5), dialyzed for 3 days and freeze-dried to finally obtain linear aliphatic BPs (82%). Carboxylic acid precursor: 4-aminobutyric acid. RMN ^{31}P (80.9 MHz, H_3PO_4/D_2O): 18.47. RMN ^1H (500 MHz, D2O): 3.046 (m, 2H), 2.017 (m, 4H). I.R. (KBr): 1540, 1172, 1052 cm^{-1};

All chemicals are purchased from Sigma-Aldrich, St Louis, MO.

Au@alendronate NPs synthesis

The procedure was adapted from previous study [3] instead of the HMBP reducing/ligands agent is replaced by alendronate. Two precursor solutions were prepared. Solution A consisted in 1 mL of 20 mM chloroauric acid ($HAuCl_4 \cdot 3H_2O$, +99.9%, Sigma-Aldrich, St Louis, MO). Solution B consisted in 2 mL of alendronate dissolved in Milli-Q water at pH adjusted at 10 with NaOH. Solution A is added to 17 mL of Milli-Q water in a round bottom flask equipped with a condenser. The solution was brought to the boil while being stirred. Solution B was added leading to a red color after 10 minutes, denoting for the apparition of nanoparticles. pH is adjusted at 7. Nanoparticles were washed with Amicon® Ultra centrifugal filters (100 K).

Au@alendronate NPs characterisations

- **ICP analysis**

The average number of alendronate molecules at the surface of the particles was determined using an ICP-AES spectrometer (iCAP 6200 duo, Thermofisher). Samples were digested in a HNO_3 and HCl solution (10 mL) evaporated then solubilized in HNO_3 2% for the analysis of phosphorus concentrations.

- **TEM analysis**

10 µL of diluted Au@alendronate NPs suspension were deposited onto 200 mesh copper grids covered by a carbon film. Observation was carried out with a Philips Tecnai 12 transmission electron microscope. Images were processed with Fiji is just Image J software on 250 particles in order to determine the nanoparticles diameter.

- **Absorbance analysis**

UV-vis spectra were recorded on a Varian Cary 50 Scan UV–vis spectrophotometer.

- **Infrared spectroscopy**

The grafting of the molecules at the surface of the particles was confirmed with Fourier transformed infrared spectroscopy (FTIR) analysis. Spectra were recorded through the form of thin KBr pellets on a Thermo Scientific Nicolet 680 FTIR.

- **Hydrodynamic diameter and zeta potential**

For hydrodynamic diameter and zeta potential measurements, a Zetasizer Nano-ZS (Malvern, U.K.) was used. The general purpose analysis model was used to convert the Dynamic light scattering (DLS) data into a size distribution (in volume) and the Schmoluchowski model to calculate the zeta potential. Measurements were made with nanoparticles solubilized in water at pH adjusted at 7.4 with 0.1 M NaOH solution to be representative of physiological conditions.

Photothermia in aqueous dispersion

Photothermal measurements were made in 1.5 mL Eppendorf tubes containing 100 µL of aqueous samples. Concentrations were adjusted between [Au] = 0.125 mM to [Au] = 2 mM.

Each sample was illuminated with a 680 nm laser (Laser Components S.A.S France) positioned 4.5 cm above at 1.7 W/cm^2 (0.78 A). The surface of the sample reached by the laser is 0.38 cm^2. The temperature was recorded with an infrared thermal imaging camera (FLIR SC7000) in real time and processed with the ALTAIR software. The heating was quantified with the plateau temperature (measured after 5 min of exposure), directly provided by the thermal IR measurements, and with the specific absorption rate (SAR), meaning the power dissipated per unit mass of gold (W·g^{-1}).

SAR was calculated using the following equation:

$$SAR = \frac{C.m_s}{m_{Au}}.\frac{\mathrm{d}T}{dt}$$

with C is the specific heat capacity of the sample (C_{water} = 4.185 J/g/K), m_{Au} is the total mass of gold in the sample (g), m_s is the total mass of the sample (g) and dT/dt is the temperature increase at the initial linear slope (30 s).

Cytotoxicity assay

PC-3 cells were purchased from ATCC and cultured in Dulbecco's modified Eagle's medium (DMEM) supplemented with 10% fetal bovine serum (FBS) and 1% penicillin-streptomycin (PS) at 37 °C with 5% CO_2. Au@Alendronate NPs were internalized by PC3 cells in a 96-well plate for 48 hours. Five conditions were compared. Firstly, cells were kept in culture for 48 hours as control condition. Secondly, culture medium was enriched with alendronate molecules, with concentrations ranging from 10^{-9} to 10^{-1} M of alendronate. Thirdly, culture medium was enriched with Au@Alendronate NPs at concentrations comprised between 10^{-2} and 10^{-9} M. Fourthly, cells were exposed to a laser 680 nm at 1 W/cm^2 as laser only control cells. Finally, cells supplemented with Au@alendronate NPs were exposed to a laser 680 nm at 1.7 W/cm^2. After 48 hours, the culture medium was replaced by 200 µL of white DMEM without phenol red, supplemented with 10% Alamar Blue. After 2 hours the plate was analysed with a multimode plate reader (Enspire Perkin Elmer) at an excitation wavelength of 570 nm and a fluorescence detection at 585 nm. Metabolic activity was determined by comparison with control cells.

Supplementary Figures

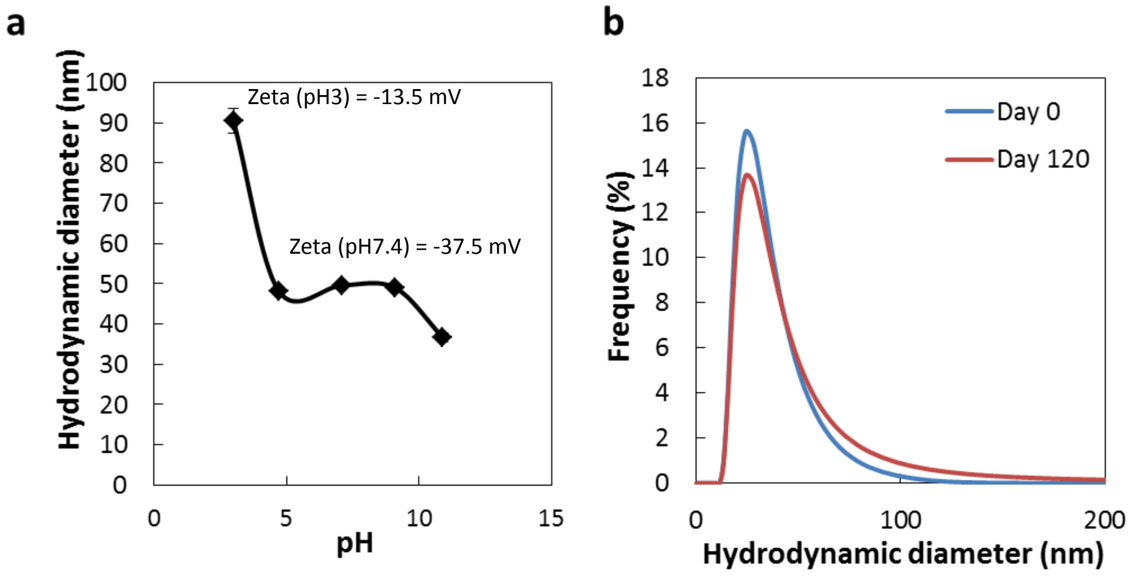

Figure S1: Stability of Au@alendronate: Hydrodynamic diameter of Au@alendronate determined by DLS measurement (a) as a function of pH (b) according to synthesis age (comparison of the diameter on the day of the synthesis and after 120 days).

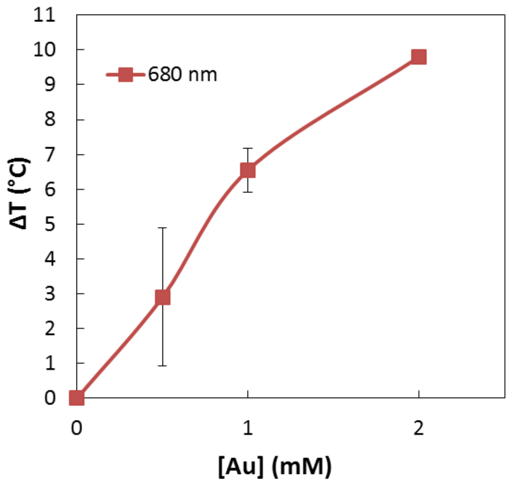

Figure S2: Temperature elevation after 5 minutes exposure to laser laser 680 nm of the Au@alendronate according to Au concentration at 0.3 W/cm^2.

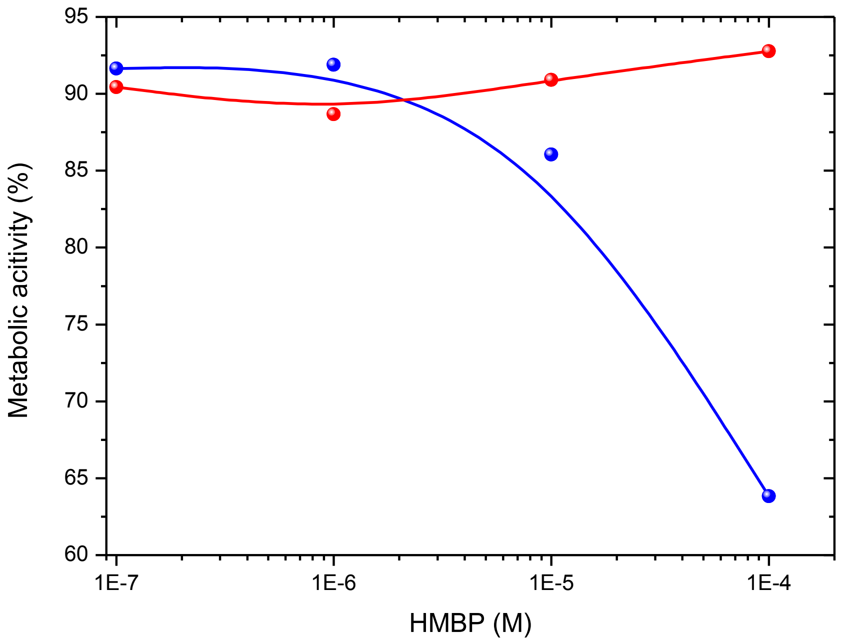

Figure S3: Metabolic activity of PC3 cells incubated with Au@alendronate NPs (blue curve) and (b) Au@HMBP-PEG NPs.

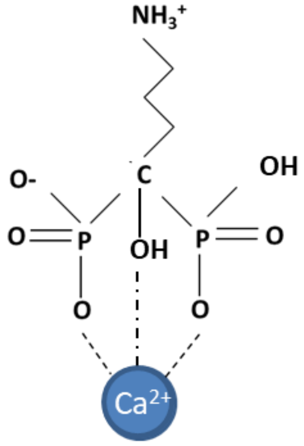

Figure S4: Alendronate molecule and its binding to hydroxyapatite (calcium phosphonate).

References

[1] Kieczykowski GR, Jobson RB, Melillo DG, Reinhold DF, Grenda VJ, Shinkai I. The Journal of Organic Chemistry 1995;60:8310-2.

[2] Guénin E, Monteil M, Bouchemal N, Prangé T, Lecouvey M. European Journal of Organic Chemistry 2007;2007:3380-91.

[3] Aufaure R, Hardouin J, Millot N, Motte L, Lalatonne Y, Guénin E. Chemistry – A European Journal 2016;22:16022-7.
